# TIE-2 Signaling Activation by Angiopoietin 2 On Myeloid-Derived Suppressor Cells Promotes Melanoma-Specific T-cell Inhibition

**DOI:** 10.3389/fimmu.2022.932298

**Published:** 2022-07-22

**Authors:** Amélie Marguier, Caroline Laheurte, Benoît Lecoester, Marine Malfroy, Laura Boullerot, Adeline Renaudin, Evan Seffar, Abhishek Kumar, Charlée Nardin, François Aubin, Olivier Adotevi

**Affiliations:** ^1^ Univ. Bourgogne Franche-Comté, INSERM, EFS BFC, UMR1098, RIGHT Interactions Greffon-Hôte Tumeur/Ingénierie Cellulaire et Génique, Besançon, France; ^2^ INSERM CIC-1431, Clinical Investigation Center in Biotherapy, Plateforme de Biomonitoring, Besançon, France; ^3^ Department of Dermatology, University Hospital of Besançon, Besançon, France; ^4^ Service Oncologie médicale, CHU Besançon, Besançon, France

**Keywords:** ANGPT2, tumor antigen, melanoma, tie-2, M-MDSCs

## Abstract

Myeloid-derived suppressor cells (MDSCs) are a heterogeneous group of immune suppressive cells detected in several human cancers. In this study, we investigated the features and immune suppressive function of a novel subset of monocytic MDSC overexpressing TIE-2 (TIE-2^+^ M-MDSC), the receptor for the pro-angiogenic factor angiopoietin 2 (ANGPT2). We showed that patients with melanoma exhibited a higher circulating rate of TIE-2^+^ M-MDSCs, especially in advanced stages, as compared to healthy donors. The distribution of the TIE-2^+^ M-MDSC rate toward the melanoma stage correlated with the serum level of ANGPT2. TIE-2^+^ M-MDSC from melanoma patients overexpressed immune suppressive molecules such as PD-L1, CD73, TGF-β, and IL-10, suggesting a highly immunosuppressive phenotype. The exposition of these cells to ANGPT2 increased the expression of most of these molecules, mainly Arginase 1. Hence, we observed a profound impairment of melanoma-specific T-cell responses in patients harboring high levels of TIE-2^+^ M-MDSC along with ANGPT2. This was confirmed by *in vitro* experiments indicating that the addition of ANGPT2 increased the ability of TIE-2^+^ M-MDSC to suppress antitumor T-cell function. Furthermore, by using TIE-2 kinase-specific inhibitors such as regorafenib or rebastinib, we demonstrated that an active TIE-2 signaling was required for optimal suppressive activity of these cells after ANGPT2 exposition. Collectively, these results support that TIE-2^+^ M-MDSC/ANGPT2 axis represents a potential immune escape mechanism in melanoma.

## Background

Myeloid-derived suppressor cells (MDSCs) are a heterogeneous group of immature myeloid origin with immunosuppressive properties. Physiologically, MDSCs play a fundamental role in the resolution of inflammation and the maintenance of immune homeostasis (1). However, MDSCs are massively accumulated in pathological conditions such as inflammation or cancer (2, 3). These cells represent one critical immune escape mechanism developed by tumor, and evidence supports the detrimental role of tumor-induced MDSC in many cancers (2, 4–6). M-MDSC are also involved in metastasis formation and treatment resistance (4, 7).

Although there are heterogeneous populations, MDSCs are classified into two subtypes, monocytic (M-MDSC) and polymorphonuclear (PMN-MDSC), which resemble monocyte and neutrophil, respectively (6). M-MDSCs are characterized by the expression of CD11b, CD14, CD33, and HLA-DR^−/low^, while PMN-MDSCs are characterized by the expression of CD11b, CD15, CD33, and CD66b (6, 8). These suppressive cells are involved in the tumor escape to immune attack through several mechanisms (9) such as the production of inhibitory cytokines (10, 11), Arginase 1 (Arg1) (12), and reactive oxygen species (2) and the regulation of adenosine mechanism by ectonucleotidase CD39 and CD73 (13).

We recently described that a subtype of M-MDSC expresses TIE-2, a receptor of the proangiogenic factor Angiopoietin 2 (TIE-2^+^ M-MDSC) (14). TIE-2 is a tyrosine kinase receptor expressed mainly by endothelial cells, cancer cells, and some immune cells like monocytes (15, 16). This receptor has many ligands in the angiopoietin family, notably ANGPT2. ANGPT2 is expressed by endothelial cells, cancer cells, and some immune cells depending on the hypoxia context or upon stimulation by different cytokines or growth factors such as TNF-α, TGF-β, and VEGF (17–20). The ANGPT2/TIE-2 axis was implicated in angiogenesis and tumor progression (21, 22) due to its role in the permeabilization of the blood vessels and the activation of TIE-2^+^-expressing monocytes (TEMs). TEMs are present in high quantity in the tumor microenvironment and blood vessels (23). In many cancers, it has been described that TEMs suppress T-cell proliferation and are implicated in neovascularization. Moreover, the immunosuppressive functions of TEMs are enhanced by the ANGPT2 stimulation. Inhibition of TIE-2 in myeloid cells induced a decrease in tumor volume and metastasis in lung cancer (23–28). In lung cancer, we identified a high rate of TIE-2^+^ M-MDSC and ANGPT2 in the metastatic stage, which are associated with a poor prognosis. Furthermore, ANGPT2 enhances immunosuppressive functions of TIE-2^+^ M-MDSC against antitumor response (14).

In this study, we investigated the TIE-2^+^ M-MDSC in patients with melanoma and their suppressive function against tumor-specific T cells. We also analyzed the expression of different proteins implicated in the immunosuppressive function of M-MDSC.

## Methods

### Patients

A total of 156 patients with melanoma were included at the University Hospital of Besançon (Besançon, France) between October 2011 and January 2016 in the LYTELOMEL cohort. Patients with cancer stages I to IV were enrolled before any anticancer therapy. All patients were included with informed consent in accordance with the French laws and after approval by the local and national ethics committees. The main clinical characteristics of the patients are summarized in **Supplementary Table S1**. Blood samples were collected before any anticancer therapy. Peripheral blood mononuclear cells (PBMCs) were isolated by density gradient separation on Ficoll Unisep tubes (Eurobio, Les Ulis, France) and frozen until use. Blood cells were also collected from 40 anonymous healthy donors (HDs) at the Etablissement Français du Sang (EFS, Besançon, France) with informed consent and following EFS guidelines.

### Flow Cytometry

To discriminate living from dead cells, PBMCs were first washed in 1× phosphate-buffered saline (PBS) (Gibco, Grand Island, NY, USA) and stained with eFluor 506 viability dye according to the manufacturer’s instructions (eBioscience, San Diego, CA, USA).

For M-MDSC analysis, samples were surface-stained in the dark for 30 min at 4°C with a mixture of the following antibodies: HLA-DR, CD14, CD33, CD11b, and TIE-2 plus lineage cocktail [(Lin) composed of CD19, CD56, and CD3].

For characterization of the MDSC phenotype, samples were surface-stained in the dark for 30 min at 4°C with different antibodies: HLA-DR, CD14, PD-L1, CD39, and CD73. Cells were fixed and permeabilized using eBioscience Foxp3/Transcription factor staining buffer set, according to the manufacturer’s instructions. After permeabilization, antibodies against IL-10, LAP, Arg1, NOS2, or COX2 were added for 30 min at 4°C and washed.

Samples were acquired on a FACS BD Canto II (BD Biosciences, San Jose, CA, USA) and analyzed with KALUZA analysis software (Beckman Coulter, Brea, CA, USA).

### ANGPT2 Measurement

The patients’ serum was collected and frozen until use. ANGPT2 was measured in patients’ serum by ELISA assay (R&D Systems, Minneapolis, MN, USA) according to the manufacturer’s instructions. The values were represented in pg/ml.

### Synthetic Peptides

A previously described mixture of eight pan MHC class II-restricted peptides derived from human telomerase (hTERT) (29, 30) and a mixture of overlapping 15-mer peptides derived from NY-ESO1 were used to analyze circulating CD4^+^ T-cell response against tumor-associated antigens (TAAs). For studying CD8^+^ T-cell response, a mixture of 12 MHC class I-restricted peptides derived from hTERT and a mixture of 5 peptides derived from NY-ESO1 were used.

hTERT MHC class II-restricted derived peptides were purchased from JPT (Berlin, Germany) (purity >80%), and NY-ESO1 MHC class II-restricted derived peptides were purchased from CTL (Cellular Technology Ltd., Shaker Heights, OH, USA). TERT and NY-ESO1 MHC class I-restricted peptides were purchased from ProImmune (Oxford, UK) (purity > 90%). To assess antiviral T-cell immunity, peptide mixtures derived from influenza virus, Epstein–Barr virus, and cytomegalovirus were used (PA-CEF-001).

### Assessment of Spontaneous T-Cell Responses Against Tumor-Associated Antigens by IFN-γ ELISpot

T-cell responses were assessed by IFNγ ELISpot assay after a short *in vitro* stimulation as described previously (29, 30). For *in vitro* stimulation, at day 0, Ficoll-isolated PBMCs were plated at 1.10^6^ cells/well for 6 days in 48-well plates with different peptide mixtures derived from hTERT and NY-ESO-1: 5 µg/ml of hTERT HLA class II, 1 µg/ml of hTERT HLA class I, or 1µg/ml of NY-ESO1. Recombinant interleukins IL-7 (5 ng/ml; PeproTech, Cranbury, NJ, USA) and IL-2 (20 UI/ml; Novartis, Basel, Switzerland) were added on days 1 and 3, respectively. On day 7, specific T-cell responses were measured by IFNγ ELISpot according to the manufacturer’s instructions (Diaclone, Besançon, France). Briefly, cells were incubated at 1.10^5^ cells/well in X-Vivo 15 medium (Lonza, Basel, Switzerland) in a 96-well ELISpot plate with the relevant peptides for 15 h. Cells cultured with medium and phorbol myristate acetate (PMA; 1 ng/ml)/ionomycin (500 ng/ml) were used as negative and positive controls, respectively. Spots were revealed, and spot-forming cells were counted using the C.T.L Immunospot System (Cellular Technology Ltd). Responses were considered positive when IFNγ spot numbers were twice those of the medium control and >10.

### ANGPT2/TIE-2^+^ Axis *In Vitro* Inhibition Assay

PBMCs from melanoma patients with TIE-2^+^ M-MDSC were assessed for T-cell response in the absence or presence of ANGPT2. Briefly, T-cell responses were evaluated by IFN-γ ELISpot, as described above. To analyze ANGPT2/TIE-2^+^ axis inhibition, 300 ng/ml of recombinant ANGPT2 was added on days 0 and 3 of the *in vitro* stimulation. In some cases, TIE-2 inhibitors—5 nM of rebastinib (MedChemExpress, Monmouth Junction, NJ, USA; DCC-2036), 50 nM of Regorafenib (Selleck, Munich, Germany; SE-S1178), or tyrosine kinase inhibitor 10 nM of dasatinib (Sigma-Aldrich, St. Louis, MO, USA; SML2589)—were added to the culture at days 0 and 3.

### Real-Time Quantitative Reverse Transcriptase PCR

Cells were collected in RLT buffer (Qiagen, Valencia, CA, USA), and total mRNAs were extracted using RNAeasy Mini Kit according to the manufacturer’s instructions (Qiagen). Total mRNA was reverse transcribed using the TaqMan gene expression assay for IL-10 (Hs00961622_m1), TEK (Hs00945150_m1), STAT3 (Hs00374280_m1), CD39 (Hs00969556_m1), CD73 (Hs00159686_m1), and Arg1 (Hs00163660_m1) (Thermo Fisher Scientific, Waltham, MA, USA) and the CFX96 Real-Time PCR Detection System (Bio-Rad, Hercules, CA, USA).

### Statistical Analysis

Without further indication, the data are presented as the mean and their associated SD. For two-group comparisons, the non-parametric Student’s t-test was used. For the survival analysis, the threshold values were calculated with the Restricted Cubic Spline method: 4.85% for TIE-2^+^ M-MDSC and 439.5 pg/ml for ANGPT2. Overall survival (OS) was calculated from the date of study enrollment to the date of death from any cause. Patients known to be alive were censored at the time of their last follow-up assessment. Information about patients’ outcomes was collected up to 7 years after their inclusion. OS was estimated using the Kaplan–Meier method described using median or rate at specific time points with 95% CI and compared among the groups with the log-rank test. All analyses were performed using Prism 7 GraphPad™ Software and R software version 2.15.2 (R Development Core Team; http://www.r-project.org). All tests were two-sided, and p-values lower than 0.05 were considered statistically significant (*p < 0.05, **p < 0.01, ***p < 0.001, ****p < 0.0001).

## Results

### Accumulation of TIE-2^+^ Monocytic Myeloid-Derived Suppressor Cells in Patients With Advanced Melanoma

**Figure 1:**
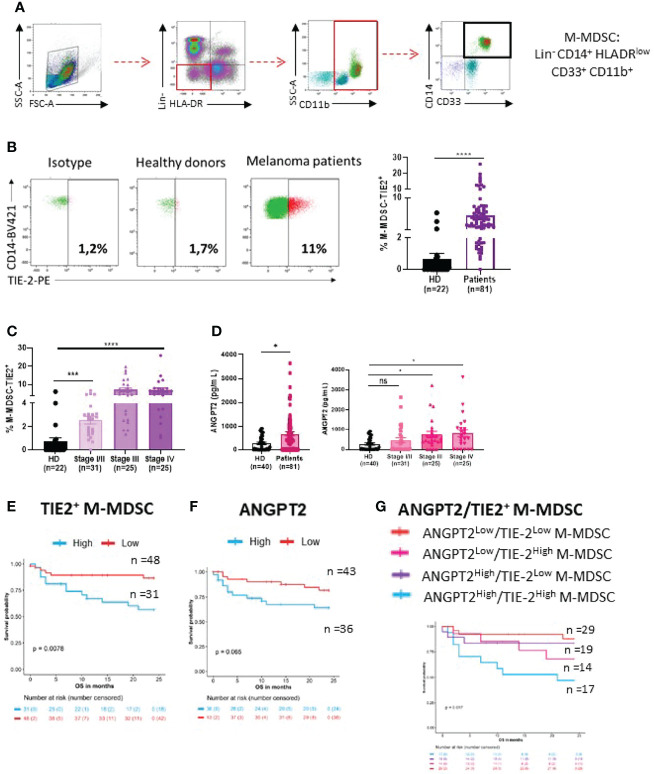
Distribution of TIE-2^+^ M-MDSC and ANGPT2 in melanoma patients. **(A)** Gating strategy of M-MDSC (Lineage^−^, HLA-DR^low^, CD11b^+^, CD33^+^ and CD14^+^). **(B)** Left, representative dot plot of TIE-2 expressed on M-MDSC from 1 healthy donor (HD) versus 1 melanoma patient gated with isotype; right, percentage of TIE-2^+^ M-MDSC in HD (n = 22) and in patients (n = 81) (Student’s t-test, ****p < 0.0001). **(C)** Percentage of TIE-2^+^ M-MDSC according to melanoma disease stages (Student’s t-test, ***p<0.001, ****p < 0.0001). **(D)** Left, level of ANGPT2 serum measured in HD (n = 22) and patient (n = 81); right, according to the disease stage (Student’s t-test, *p < 0.01). **(E–G)** Association between overall survival (OS) and percentage of TIE-2^+^ M-MDSC **(E)**, ANGPT2 concentration **(F)**, and the combination of both parameters **(G)**. **(E)** Kaplan–Meier curves according to percentage of TIE-2^+^ M-MDSC in overall population (p = 0.0079). Thresholds were determined according to the restricted cubic spline method (4.85%). **(F)** Kaplan–Meier curves according to concentration of ANGPT2 in overall population (p = 0.062). Thresholds were determined according to the restricted cubic spline method (439.5 pg/ml). **(G)** Patients were classified into 4 distinct groups according to the level of TIE-2^+^ M-MDSC and ANGPT2 concentration. Kaplan–Meier curves for the 4 groups in overall population (p = 0.017). ns, no signigicant.

TIE-2 expressing M-MDSCs (TIE-2^+^ M-MDSC) were analyzed by flow cytometry in peripheral blood from melanoma patients (n = 81) and HDs (n = 22) as control. The phenotype of M-MDSC was Lineage^−^ (CD3^−^, CD19^−^, and CD56^−^), HLA-DR^low^, CD11b^+^, CD33^+^, and CD14^+^, as previously described (31) (**Figure 1A**). A higher circulating rate of TIE-2^+^ M-MDSC was observed in melanoma patients than in HDs (7.3% vs 1.5%, p < 0.0001), and this rate was significantly more increased in advanced stages (**Figures 1B, C** and **Supplementary Table 1**). Moreover, most advanced patients displayed high levels of circulating TIE-2^+^ M-MDSC (>5%) as compared to stage I/II patients (**Figure 1C**). Accordingly, a higher amount of serum ANGPT2, the ligand of TIE-2, was shown in melanoma patients and especially in advanced stages (**Figures 1D, E**). As expected, we found that a high level of TIE-2^+^ M-MDSC (>4.85%) was associated with lower OS as compared to patients with TIE-2^low^ M-MDSC (55% vs 80% alive at 24 months, p = 0.0078) (**Figure 1E**). A higher amount of ANGPT2 was also associated with poor outcomes in melanoma (70% vs 87% alive at 24 months, p = 0.065), in line with the literature (31–34) (**Figure 1F**). Accordingly, patients exhibiting both TIE-2^+^ M-MDSC/ANGPT2^high^ profiles had a bad prognosis, and their median OS was 7 months versus not reached in the group TIE-2^+^ M-MDSC/ANGPT2^low^ (p = 0.019) (**Figure 1G**). This negative impact associated with a high TIE-2^+^ M-MDSC/ANGPT2 environment was mainly related to advanced stages since patients who belonged to stages I and II often had an overall lower level of circulating TIE-2^+^ M-MDSC or ANGPT2 (**Supplementary Figures 1A, B**).

### A High Rate of TIE-2^+^ Monocytic Myeloid-Derived Suppressor Cells is Associated with An Impairment of Melanoma-Specific T-Cell Responses

**Figure 2:**
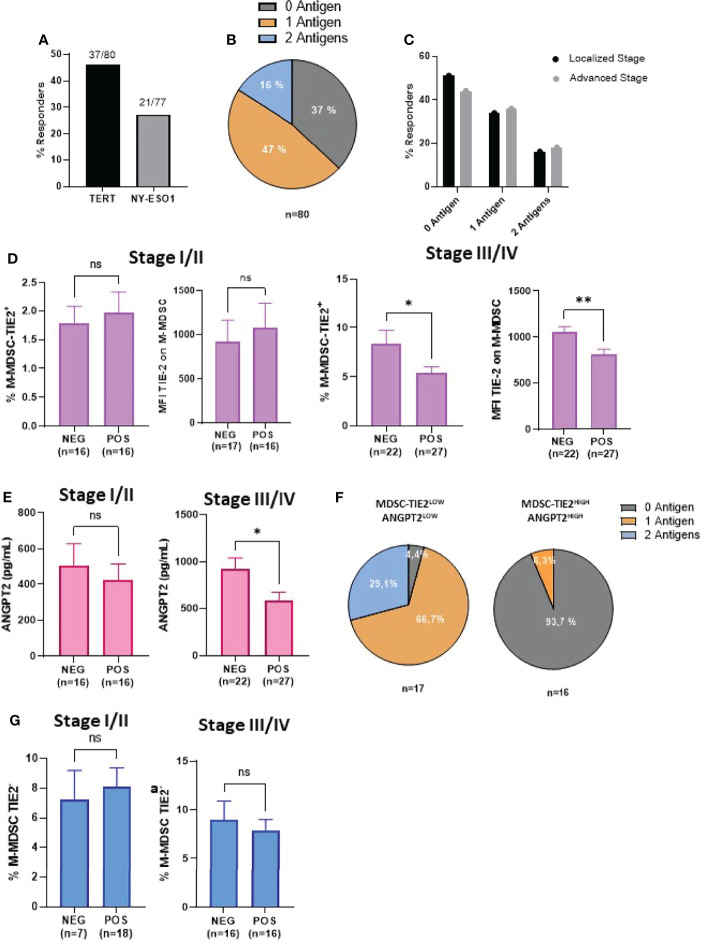
Correlation between TIE-2^+^ M-MDSC/ANGPT2 and antitumor-specific T-cell responses in melanoma patients. **(A)** Frequency of patients exhibiting spontaneous T-cell responses against TERT (37/80) or NY-ESO1 (21/77) antigens by IFN-γ ELISpot assay. **(B)** Distribution of patients according to their antitumor responding responses against 0, 1, or 2 antigens (n = 80). **(C)** Responders’ frequency to 0, 1, or 2 antigens between localized or advanced stages. **(D)** TIE-2^+^ M-MDSC percentage and TIE-2 MFI expression according to antitumor T-cell response negative (NEG) or positive (POS) in localized stage (I–II) (left) and in advanced stage (III–IV) (right) (Student’s t-test, * p < 0.1). **(E)** ANGPT2 concentration according to antitumor T-cell response negative (NEG) or positive (POS) in localized stage (I–II) (left) and in advanced stage (III–IV) (right) (Student’s t-test, *p < 0.1, **p < 0.01). **(F)** Distribution of antitumor T-cell responses in TIE-2^high^ M-MDSCs, ANGPT2^high^ (n = 16) vs TIE-2^low^ M-MDSCs, and ANGPT2^low^ (n = 17). **(G)** TIE-2^neg^ M-MDSC percentage according to antitumor T-cell response negative (NEG) or positive (POS) in localized stage (I–II) (left) and in advanced stage (III–IV) (right) (Student’s t-test, *p < 0.1). ns, no signigicant.

The above results suggest that the deleterious effect of TIE-2^+^ M-MDSC may be related to their inhibitory effect on antitumor T-cell responses, as previously described (14). To investigate this purpose, we measured spontaneous T-cell responses directed against a mixture of peptides derived from hTERT and NY-ESO-1, two tumor antigens highly expressed in melanoma or with virus-derived peptides used as non-tumor antigens (36–38). The frequencies of responder patients to hTERT and NY-ESO1 measured by INF-γ ELISpot assay were 46% (37/80) and 27.3% of patients (21/77), respectively (**Figure 2A**). Overall, 47% of patients responded to at least one antigen, and 16% responded against the two antigens. The frequency of immune responders against one and two melanoma-associated antigens was equivalent regardless of the melanoma stage (**Figures 2B, C**). In contrast to stage I/II patients, we found that in the advanced stage III/IV group, non-responder patients exhibited a high level of TIE-2^+^ M-MDSC than responder patients (8.4 vs 5.4%, p < 0.03). A similar trend was found with mean fluorescence intensity (MFI) value of TIE-2 expression on M-MDSC: mean MFI 816 vs 1,001 in immune responders and non-responders, respectively (** p < 0.01) (**Figure 2D**). Similar trends were made with the ANGPT2 level in these two groups of localized or advanced patients (**Figure 2E**). As a result, we found that the majority of patients (>90%) had functional melanoma-specific T-cell responses in the group of patients with TIE-2^+^ M-MDSC/ANGPT2^low^ profile, whereas only 6% of patients with TIE-2^+^ M-MDSC/ANGPT2^high^ had a preexisting antitumor T-cell response (**Figure 2F**).

Of note is that no obvious relationship was shown between the antiviral T-cell responses and TIE-2^+^ M-MDSC or ANGPT2 (**Supplementary Figures 2A–C**). Furthermore, no correlation was observed between TIE-2^neg^ M-MDSC and melanoma-specific T-cell response in this population (**Figure 2G**). Thus, a high level of TIE-2^+^ M-MDSC/ANGPT2 in peripheral blood is associated with impaired antitumor T-cell responses in advanced melanoma, suggesting that this proangiogenic pathway may suppress tumor-specific T cell in melanoma.

### ANGPT2 Increases Immune Suppressive Features of TIE-2^+^ Monocytic Myeloid-Derived Suppressor Cells

**Figure 3:**
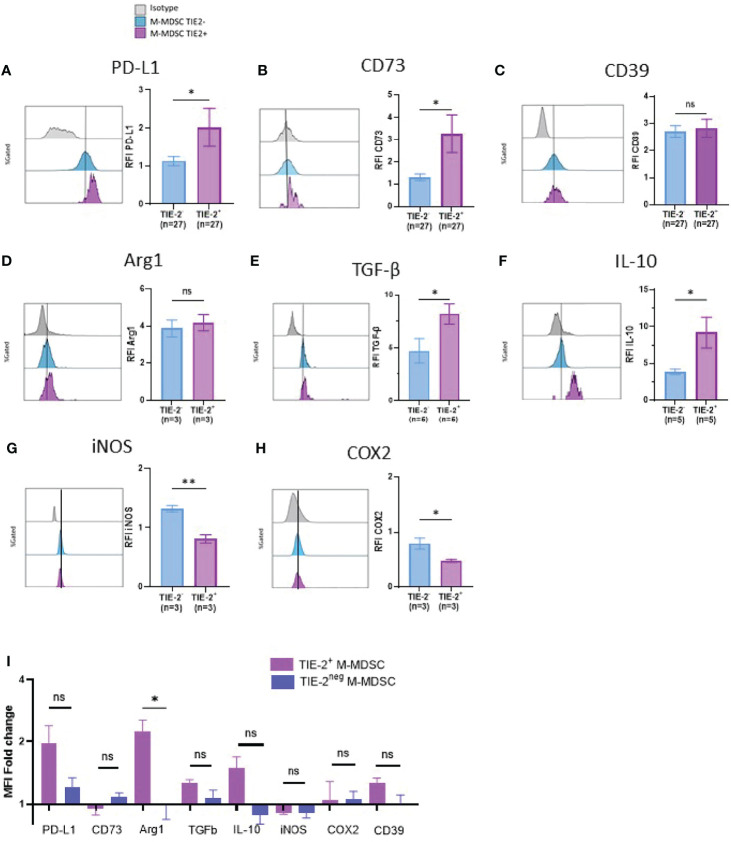
Immune suppressive factors of TIE-2^+^ M-MDSC. **(A–H)** Expression of different proteins (PD-L1, CD39, CD73, Arg1, TGF-β, IL-10, iNOS, and COX2) was studied in TIE-2^+^ M-MDSC (TIE-2^+^) and TIE-2^−^ M-MDSC (TIE-2^−^). Left, histogram overlay from one representative patient of the mean fluorescence intensity (MFI) of different proteins; right, relative fluorescence intensity (RFI) of these proteins (Mann–Whitney test, *p < 0.1). **(I)** TIE-2^+^ M-MDSC enriched melanoma patients’ peripheral blood mononuclear cells (PBMCs) were stimulated or not with 300 ng/ml of ANGPT2 overnight, and the expression of the different immunosuppressive proteins was analyzed by flow cytometry. Histograms of the MFI fold change. ns, no signigicant.

To scrutinize the inhibitory role of the TIE-2^+^ M-MDSC/ANGPT2 axis, we analyzed the expression of inhibitory pathways related to suppressive cells (2, 10–13, 39) on TIE-2^+^ M-MDSCs using flow cytometry. We showed that TIE-2^+^ M-MDSCs expressed a higher level of PD-L1 and CD73 but not of CD39 than TIE-2^neg^ M-MDSC (**Figures 3A–C**). Next, we performed intracellular staining of suppressive factors such as Arg1, IL-10, and TGF-β on TIE-2^+^ versus TIE-2^neg^ M-MDSC. Although increased IL-10 and TGF-β expression levels were detected on TIE-2^+^ M-MDSC, no obvious change was observed for Arg1 expression (**Figures 3D–F**). In contrast, the expression of iNOS and COX2 appeared lower in TIE-2^+^ as compared to TIE-2^neg^ M-MDSC (**Figures 3G, H**). We demonstrated that the *in vitro* exposition of TIE-2^+^ M-MDSC to recombinant ANGPT2 stimulation enhanced the expression of most of these inhibitory pathways, mainly PD-L1 and Arg1, but did not influence the expression of iNOS and COX2 in TIE-2^+^ M-MDSC. As expected, ANGPT2 had no effect on TIE-2^neg^ M-MDSC (**Figure 3I** and **Supplementary Figure 3A**), suggesting the upregulation of these suppressive factors involved in TIE-2 signaling. Furthermore, we showed that ANGPT2 treatment of M-MDSC TIE-2 also upregulated transcripts such as Arg1, TIE-2, and CD39 in line with protein level (**Supplementary Figure 3B**). Thus, ANGPT2 enhances suppressive pathways in TIE-2^+^ M-MDSC.

### ANGPT2/TIE-2 Signaling on Monocytic Myeloid-Derived Suppressor Cells Inhibits IFN-y Production by Melanoma-Specific T Cells

**Figure 4:**
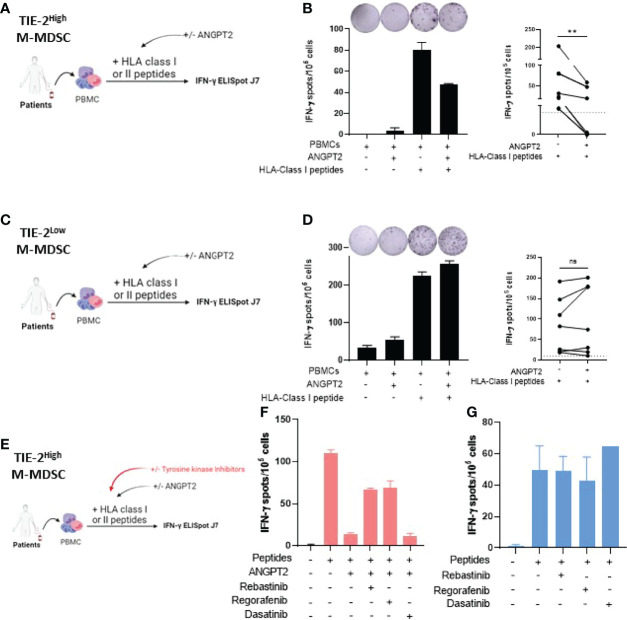
Effect of ANGPT2/TIE-2 signaling on M-MDSC on antitumor responses. **(A)** Peripheral blood mononuclear cells (PBMCs) from melanoma patients with TIE-2^high^ M-MDSC were stimulated with TERT class I peptides in presence or not of 300 ng/ml of ANGPT2, and an IFN-γ ELISpot assay was performed. **(B)** Left, IFN-γ T-cell responses of one representative example of patient; right, histograms from 6 patients (Wilcoxon test, **p < 0.01). **(C)** PBMCs from melanoma patients with TIE-2^low^ M-MDSC were stimulated with TERT class I peptides in presence or not of 300 ng/ml of ANGPT2, and an IFN-γ ELISpot assay was performed. **(D)** Left, IFN-γ T-cell responses of one representative example of patient; right, histograms from 8 patients (Wilcoxon test, **p < 0.01). **(E)** PBMCs from melanoma patients with TIE-2^high^ M-MDSC were stimulated with TERT class I or class II peptides in presence or not of ANGPT2 and in presence or not of TIE-2 inhibitors, and then an IFN-γ ELISpot was performed. **(F)** Histograms of IFN-γ T-cell responses in presence of ANGPT2 (n = 6). **(G)** Histograms of IFN-γ T-cell responses in the absence of ANGPT2 (n = 6). ns, no signigicant. ns, no signigicant.

To study the involvement of ANGPT2/TIE-2^+^ M-MDSC in the inhibition of antitumor T-cell function, we first performed *in vitro* stimulation on PBMCs from patients with elevated levels of TIE-2^+^ M-MDSCs in the presence or not of recombinant ANGPT2 (**Figure 4A**). Immune responder patients were selected, and hTERT and NY-ESO1 derived HLA class I binding peptides were used for T-cell stimulation. As shown in **Figure 4B**, IFN-γ production of tumor-specific CD8 T-cell responses significantly decreased in the presence of ANGPT2. Similar results were obtained against tumor-specific CD4 T-cell response (**Supplementary Figure 4**). In contrast, ANGPT2 had no effect on tumor-specific T-cell response in the context of TIE-2^low^ M-MDSC patients (**Figures 4C, D**).

To demonstrate that ANGPT2 acts through TIE-2^+^ signaling, we performed similarly *in vitro* stimulation experiments in the presence or not of TIE-2 kinase inhibitors such as rebastinib and regorafenib (40–42). Dasatinib, a BCR-ABL kinase inhibitor, was used as a control (**Figure 4E**). The results showed that, in contrast to dasatinib, the addition of both regorafenib and rebastinib effectively restored the IFN-γ production by tumor-specific T cells inactivated by ANGPT2 exposition (**Figure 4F**). In the absence of ANGPT2, the TIE-2 inhibitors did not affect IFN-γ production by melanoma-specific T cells (**Figure 4G**). Thus, TIE-2^+^ signaling activation on M-MDSC through ANGPT2 binding inhibits IFN-γ secretion by tumor-reactive T-cells.

## Discussion

In this study, we showed that circulating TIE-2^+^ M-MDSC in melanoma patients displayed high immunosuppressive patterns than the TIE-2^neg^ counterpart and were accumulated in advanced stages. The high rate of these cells was associated with low melanoma-specific T-cell responses, and ANGPT2 increases the ability of TIE-2^+^ M-MDSC to suppress melanoma-specific T-cell functions. We demonstrated that the involvement of TIE-2 kinase activation in T-cell inhibition is mediated by TIE-2^+^ M-MDSC. As a result, a high level of TIE-2^+^ M-MDSC together with ANGPT2 in peripheral blood was associated with a very poor prognosis.

These results confirmed the negative impact on clinical outcomes in melanoma as we previously reported in lung cancer patients (14). This population of TIE-2^+^ M-MDSC is distinct from the previously described TEMs, which are characterized by the expression of CD16^+^, CD14^low^, HLADR^+^, and CD62L^−^ (43). These cells were previously found in the tumor microenvironment and peripheral blood (23).

Here, we found that TIE-2 expression on M-MDSC enhances the suppressive features of M-MDSC such as the overexpression of PD-L1, CD73, IL-10, and TGF-β, which are proteins involved in their inhibitory roles (44–46). The addition of ANGPT2 also enhances the expression of many of these inhibitory pathways including Arg1, which is known to promote essential amino acid l-arginine depletion and in turn suppresses T cells. This effect was also described in TEMs, which overexpressed IL-10 after ANGPT2 stimulation (25). In contrast, the expression of other immune-suppressive molecules, such as iNOS and COX2, was not influenced by TIE-2 kinase signaling. Similar observations were previously reported in the case of TEMs in a mouse tumor model by using transcriptomic analysis. The authors showed a high level of Arg1 transcript but lower COX2 and iNOS in TEMs (47).

These phenotypic changes of M-MDSC mediated by TIE-2 signaling after ANGPT2 stimulation toward a highly immunosuppressive role were thereby confirmed by the functional profiling. Hence, in line with our previous report (14), we showed the effect of ANGPT2 stimulation enhanced the capacity of TIE-2^+^ M-MDSC to suppress melanoma-specific T-cell function *in vitro*. This is also in accordance with the complete impairment of spontaneous anti-melanoma T-cell responses observed in melanoma patients harboring both ANGPT2 and TIE-2^+^ M-MDSC-rich blood.

Although the precise mechanism by which TIE-2 intracellular pathway mediates IFN-γ production by T cells is not yet elucidated, we demonstrated that this involved an active TIE-2 signaling, since the use of TIE-2 kinase-specific inhibitors restored this ability of tumor-specific T-cells. In our experiments, we cannot exclude the possible participation of TEMs in the inhibitory role exerted by ANGPT2 on T-cell responses. Nevertheless, our preliminary finding by using co-culture of antitumor T-cell clone with TIE-2^+^ cells sorted from PBMCs suggests that M-MDSC displayed more suppressive capacity than TEMs. However, these observations deserve future investigations.

In conclusion, this study in melanoma shows the ability of TIE-2^+^ M-MDSC to suppress antitumor T-cell function through ANGPT2 stimulation. Together with our first report, our results support that TIE-2^+^ M-MDSC/ANGPT2 signature represents a tumor escape mechanism across human cancers. Our finding also encourages combining TIE-2 inhibitors with immunotherapy in melanoma.

## Data Availability Statement

The raw data supporting the conclusions of this article will be made available by the authors upon reasonable request.

## Ethics Statement

The studies involving human participants were reviewed and approved by CPP EST-II (France). The patients/participants provided their written informed consent to participate in this study.

## Author Contributions

AM, BL, MM, LB, and AR realized experiment. All authors provided inputs on the data analyses. AM, CL. and OA wrote the first draft of the manuscript. All authors contributed to the article and approved the submitted version.

## Funding

This work was supported by La ligue contre le cancer (Grand Est, call for projets 2020, OPE= 2021-0009) and by the Région Bourgogne Franche-Comté (Grand ISIT 2019).

## Conflict of Interest

The authors declare that the research was conducted in the absence of any commercial or financial relationships that could be construed as a potential conflict of interest.

## Publisher’s Note

All claims expressed in this article are solely those of the authors and do not necessarily represent those of their affiliated organizations, or those of the publisher, the editors and the reviewers. Any product that may be evaluated in this article, or claim that may be made by its manufacturer, is not guaranteed or endorsed by the publisher.

## Supplementary Material

Supplementary Table 1 Melanoma patients’ main clinical characteristics.

Supplementary Figure 1 Overall survival of ANGPT2/TIE-2^+^ M-MDSC according to the melanoma disease stage. **(A)** Association between overall survival (OS) and the percentage of TIE-2^+^ M-MDSC according to the melanoma disease stage. Kaplan–Meier curves according to percentage of TIE-2^+^ M-MDSC in localized stage (I-II, p = 0.71) and in advanced stage (III-IV, p = 0.18). Threshold were determined according to the Restrictped Cubic Spline method (4.85%). **(B)** Association between OS and the concentration of ANGPT2. Kaplan–Meier curves according to concentration of ANGPT2 in localized stage (p = 0.33) and in advanced stage (p = 0.067). Threshold were determined according to the Restricted Cubic Spline method (439.5 pg/mL). **(C)** Patients were classified into 2 distinct groups according to the level of TIE-2^+^ M-MDSC and ANGPT2 concentration in advanced stage. Kaplan–Meier curves for the 2 groups in advanced stages (p = 0.043).

Supplementary Figure 2 Correlation between TIE-2^+^ M-MDSC/ANGPT2 and anti-viral T cell responses in melanoma patients. **(A)** TIE-2^+^ M-MDSCs percentage according to antiviral T cells response negative (NEG) or positive (POS) in localized stage (I-II) (right) and in advanced stage (III-IV) (left) (Student T test *p <0,1). **(B)** ANGPT2 concentration according to antiviral T cells response negative (NEG) or positive (POS) in localized stage (I-II) (right) and in advanced stage (III-IV) (left) (Student T test * p <0,1). **(C)** TIE-2^neg^ M-MDSCs percentage according to antiviral T cells response negative (NEG) or positive (POS) in localized stage (I-II) (right) and in advanced stage (III-IV) (left) (Student T test *p <0,1)

Supplementary Figure 3 Immune suppressive factors of TIE-2^+^M-MDSC. **(A)** MFI expression of different proteins (PD-L1, CD39, CD73, Arg1, TGF-β, IL-10, iNOS and COX2) studied in TIE-2^+^ M-MDSC and in TIE-2^neg^ M-MDSC after or not exposition to ANGPT2. **(B)** mRNA expression of different gene (TIE-2, CD73, Arg1, IL-10, STAT3 and CD39) were analyzed by RT-qPCR in TIE-2^High^ and TIE-2^Low^ M-MDSC and the fold change after ANGPT2 exposition was calculated.

Supplementary Figure 4 Effect of ANGPT2/TIE-2 signaling on M-MDSC on anti-tumor responses. PBMCs from melanoma patients with TIE2^High^ M-MDSC were stimulated with TERT class II peptides in presence or not of 300 ng/mL ANGPT2 and an IFN-γ ELISpot assay was performed. IFN-γ T cell responses, in left, one representative example of patient; in right, histograms from 6 patients respectively (Wilcoxon test **p< 0,01; *p<0,1).
