# Supplementary figures and images for: TIE-2 Signaling Activation by Angiopoietin 2 On Myeloid-Derived Suppressor Cells Promotes Melanoma-Specific T-cell Inhibition
Source: Front Immunol. 2022 Jul 22;13:932298. doi: 10.3389/fimmu.2022.932298 (PMC9353943; doi:10.3389/fimmu.2022.932298)

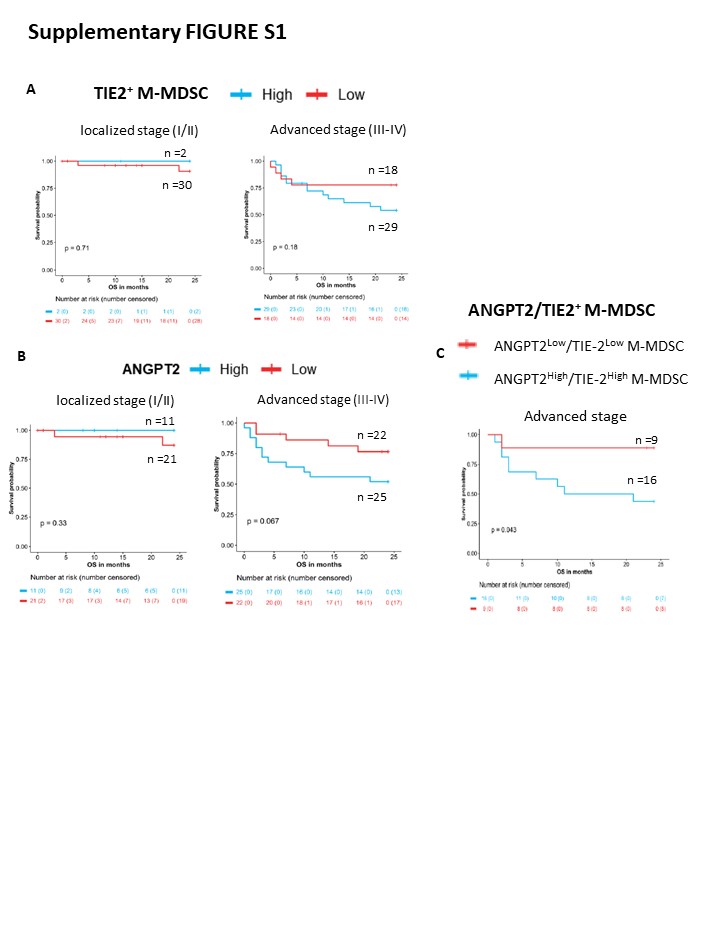

Supplement: Supplementary Table 1 — Melanoma patients’ main clinical characteristics. [file Image_1.jpeg]

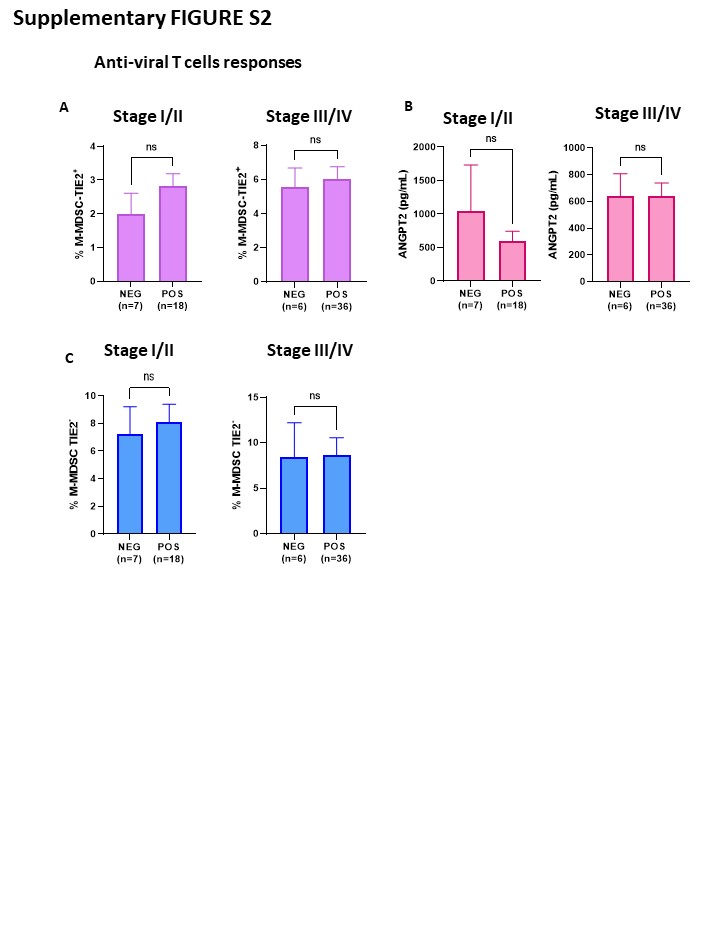

Supplement: Supplementary Figure 1 — Overall survival of ANGPT2/TIE-2+ M-MDSC according to the melanoma disease stage. (A) Association between overall survival (OS) and the percentage of TIE-2+ M-MDSC according to the melanoma disease stage. Kaplan–Meier curves according to percentage of TIE-2+ M-MDSC in localized stage (I-II, p = 0.71) and in advanced stage (III-IV, p = 0.18). Threshold were determined according to the Restrictped Cubic Spline method (4.85%). (B) Association between OS and the concentration of ANGPT2. Kaplan–Meier curves according to concentration of ANGPT2 in localized stage (p = 0.33) and in advanced stage (p = 0.067). Threshold were determined according to the Restricted Cubic Spline method (439.5 pg/mL). (C) Patients were classified into 2 distinct groups according to the level of TIE-2+ M-MDSC and ANGPT2 concentration in advanced stage. Kaplan–Meier curves for the 2 groups in advanced stages (p = 0.043). [file Image_2.jpeg]

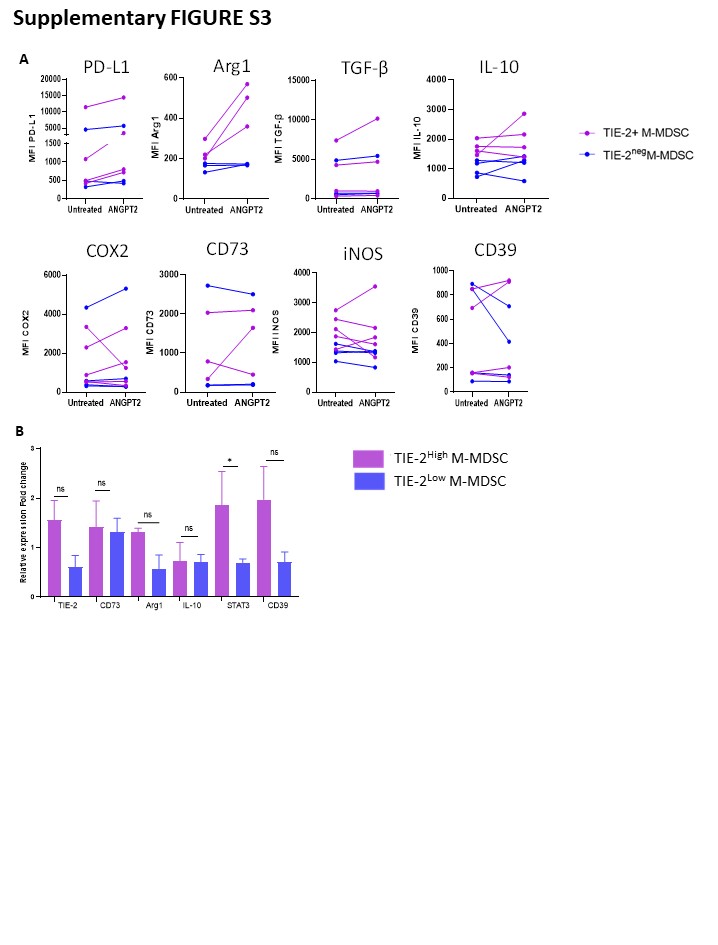

Supplement: Supplementary Figure 2 — Correlation between TIE-2+ M-MDSC/ANGPT2 and anti-viral T cell responses in melanoma patients. (A) TIE-2+ M-MDSCs percentage according to antiviral T cells response negative (NEG) or positive (POS) in localized stage (I-II) (right) and in advanced stage (III-IV) (left) (Student T test *p <0,1). (B) ANGPT2 concentration according to antiviral T cells response negative (NEG) or positive (POS) in localized stage (I-II) (right) and in advanced stage (III-IV) (left) (Student T test * p <0,1). (C) TIE-2neg M-MDSCs percentage according to antiviral T cells response negative (NEG) or positive (POS) in localized stage (I-II) (right) and in advanced stage (III-IV) (left) (Student T test *p <0,1) [file Image_3.jpeg]

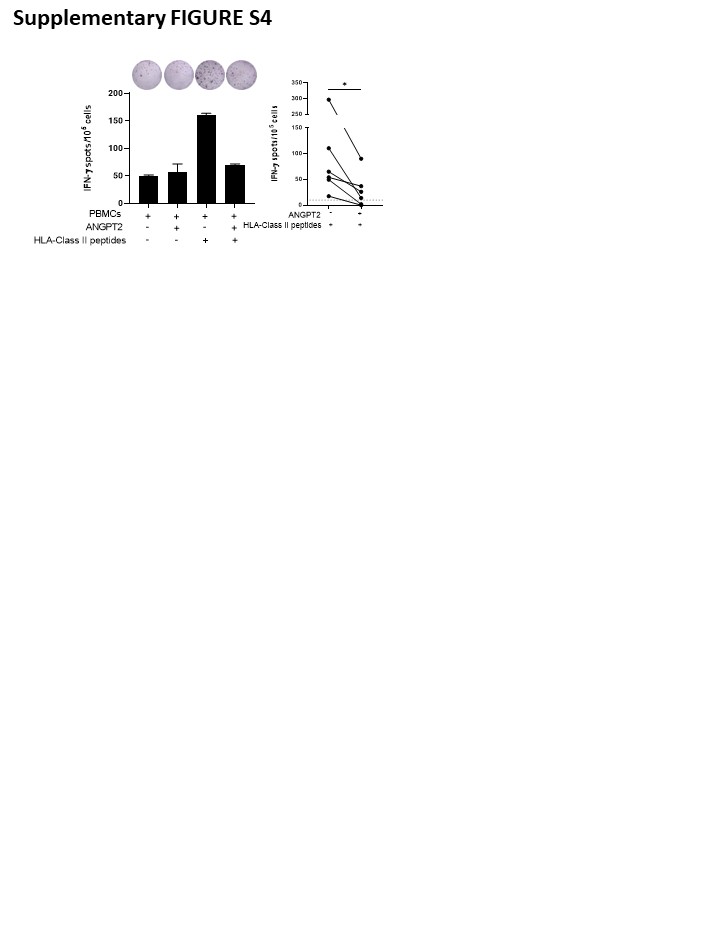

Supplement: Supplementary Figure 3 — Immune suppressive factors of TIE-2+M-MDSC. (A) MFI expression of different proteins (PD-L1, CD39, CD73, Arg1, TGF-β, IL-10, iNOS and COX2) studied in TIE-2+ M-MDSC and in TIE-2neg M-MDSC after or not exposition to ANGPT2. (B) mRNA expression of different gene (TIE-2, CD73, Arg1, IL-10, STAT3 and CD39) were analyzed by RT-qPCR in TIE-2High and TIE-2Low M-MDSC and the fold change after ANGPT2 exposition was calculated. [file Image_4.jpeg]

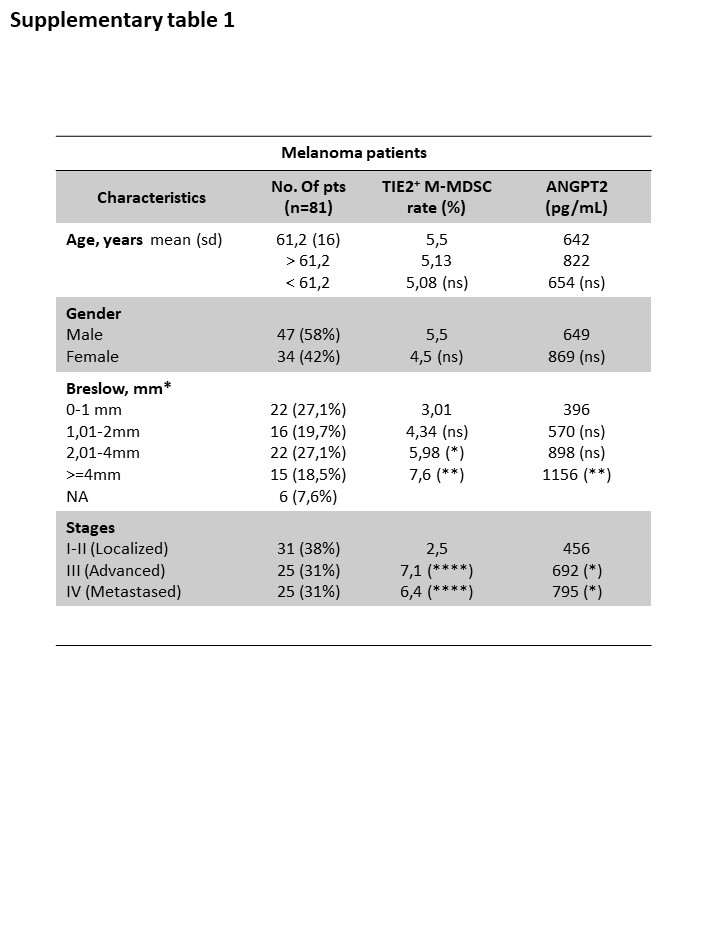

Supplement: Supplementary Figure 4 — Effect of ANGPT2/TIE-2 signaling on M-MDSC on anti-tumor responses. PBMCs from melanoma patients with TIE2High M-MDSC were stimulated with TERT class II peptides in presence or not of 300 ng/mL ANGPT2 and an IFN-γ ELISpot assay was performed. IFN-γ T cell responses, in left, one representative example of patient; in right, histograms from 6 patients respectively (Wilcoxon test **p< 0,01; *p<0,1). [file Image_5.jpeg]
